# Discomfort towards peers causes therapy-related stress in children and adolescents on daily rhGH therapy

**DOI:** 10.3389/fendo.2025.1520210

**Published:** 2025-03-13

**Authors:** Domenico Corica, Cecilia Lugarà, Chiara Ferraloro, Angela Alibrandi, Valentina La Malfa, Maria Pecoraro, Giorgia Pepe, Letteria Anna Morabito, Tommaso Aversa, Malgorzata Gabriela Wasniewska

**Affiliations:** ^1^ Department of Human Pathology of Adulthood and Childhood “G. Barresi”, Pediatric Unit, University of Messina, Messina, Italy; ^2^ Department of Economics, University of Messina, Messina, Italy

**Keywords:** growth hormone, GH deficiency, daily treatment, childhood, fear of needles, adherence, quality of life, questionnaire

## Abstract

**Introduction:**

Recombinant human growth hormone (rhGH) therapy is a long-term, daily, injective treatment, which can be burdened by poor adherence affected by several factors. In addition, chronic daily administration of rhGH may cause stress and discomfort in the child and the caregivers, independent of the degree of adherence. Aims of this study are: 1. to evaluate the degree of adherence and the presence of stress related to daily treatment with rhGH on the basis of data reported by patients and caregivers; 2. to assess which factors influence adherence to rhGH therapy and therapy-related stress.

**Methods:**

Patients on rhGH therapy for at least one year, diagnosed with isolated GH deficiency (GHD) or on rhGH therapy because small for gestational age (SGA) were included. Patients and/or caregivers were administered a questionnaire on family background, duration and management of rhGH therapy, perception of effectiveness, adherence, fear of needles, chronic therapy-related stress, discomfort towards peers related to chronic treatment.

**Results:**

Seventy patients were recruited (mean age 11.7 ± 2.86 years). Good adherence was reported in 82.9% of cases while medium/poor adherence was reported in 17% of the cases. Fear of needles was reported in 25.7% of cases and discomfort towards peers related to chronic treatment in 22.9% of cases. Therapy-related stress was declared in 41.4% of cases. At the univariate regression analysis, therapy-related stress was influenced by fear of needles (OR 2.9, 95%CI 1.05-8.97; p=0.044) and discomfort towards peers (OR 4.4, 95%CI 1.32-14.59; p=0.015). Multivariate regression analysis confirmed the significant influence of discomfort towards peers on chronic therapy-related stress (OR 4.84, 95%CI 1.30-17.99; p=0.019) independently of gender, age, pubertal stage and fear of needles.

**Conclusion:**

Discomfort towards peers related to chronic treatment was associated to therapy-related stress in childhood, despite a high degree of adherence. These factors should be identified early to plan strategies to mitigate their negative impact on the quality of life of patients treated with rhGH.

## 1 Introduction

Therapy with recombinant human growth hormone (rhGH) is a long-term, daily, injective treatment, whose benefits are not immediately appreciated by patients and their caregivers (1). Treatment with rhGH appears to be safe and relatively well tolerated (2, 3). The main goals of rhGH therapy in childhood and adolescence are to normalize the growth rate as soon as possible and to achieve a final height within normal limits and within the family target (4, 5). Adherence to therapy significantly influences the achievement of treatment goals (6, 7). Adherence can be defined as *“the extent to which a person’s behavior, with regard to taking medication, following a diet and/or executing lifestyle changes, corresponds with agreed recommendations from a health-care provider”* (8) or alternatively as “*the extent to which the patient’s behavior … coincides with clinical prescription*” (7, 9). Chronic daily rhGH therapy can be burdened by poor adherence to treatment. There are several reasons for poor adherence in pediatric age, although they are not always clearly identified in real clinical settings, including long duration of therapy, physician-patient relationship, socio-economic and educational level of caregivers, poor understanding of the mechanisms and benefits of therapy, inadequate therapeutic training, problems related to the use of injection device, difficulty in administration, fear of needles, pain related to injection (7, 10–13).

Available data report that the rate of adherence to therapy is generally good in pediatric age (>80%) (6). These findings could be partly due to the involvement of a third party in therapeutic management, i.e. parents/caregivers (14). However, the estimate of the degree of non-adherence is quite variable (from 5% to 82%) depending on the methods and definitions applied (7).

Moreover, even with a high adherence rate, children and adolescents may be particularly stressed by the daily injections, resulting in a low quality of life (QoL) compared to peers who are not receiving chronic treatment (6, 12, 15, 16). Early identification of poor adherence and/or therapy-related stress/discomfort is crucial for treatment outcomes and for planning intervention strategies aimed at improving compliance to therapy.

The aims of this study are: 1. to evaluate the degree of adherence and the presence of stress related to daily treatment with rhGH on the basis of data reported by patients and caregivers; 2. to assess which factors influence adherence to rhGH and therapy-related stress.

## 2 Materials and methods

An observational, single-center study was conducted at the Pediatric Endocrinology Centre of the University Hospital “G. Martino” of Messina, Italy, from April 2022 to March 2024. Patients were recruited during their routine six-monthly follow-up. Patients on rhGH therapy for at least one year, diagnosed with isolated GH deficiency (GHD) or on rhGH therapy because small for gestational age (SGA) were included. The exclusion criteria were: chronic disease; other hormone deficiencies; genetic syndromes; failing to attend routine follow-up checkups.

Anamnestic (including diagnosis, weight and length at birth, date of start of rhGH treatment, dosage of rhGH mg/kg/day administered), clinical and auxological parameters (including height, BMI, growth velocity, pubertal stage, bone age), and biochemical data (including IGF-1 before and during therapy) were collected at the follow-up visit. Height measurements were in a standing position using Harpenden stadiometer. Weight was recorded using a mechanical column scale with sliding counterweights. The BMI value was calculated using the formula weight (kg)/height (m^2^).

A questionnaire was administered to patients and/or caregivers (in the case of children < 6 years of age) at the six-month control visit, under the supervision of a psychologist. The questionnaire administered to patients was developed based on Smith et al.’s questionnaire (17), modified according to the current social, cultural and clinical context, after review and agreement with the team involved in the study. The questionnaire did not have a scoring system for patient responses. The questionnaire included items on family background, home therapy management and emotions related to chronic therapy, including questions on parents’ education, employment, household residence, household composition, rhGH therapy duration and dosage, who gives the injections (patient or caregivers), features of the device, previous therapy training and desire to attend one, number of missed doses in the previous six months, perception of therapy’s effectiveness, misperceptions about the consequences of missed rhGH doses, discomfort with injections, fear of needles, chronic therapy-related stress, discomfort towards peers related to chronic treatment, problems transporting vials, interest in long-acting rhGH therapy. The questionnaire did not have a scoring system for patient responses.

The intellective quotient (IQ) of the patients was assessed by the psychologist using Raven’s Progressive Matrices prior to the administration of the questionnaire to the patient and/or caregivers.

Adherence was defined as good (one missed dose per week; >86% of doses administered) or as moderate/poor (greater than one missed dose per week; <86% of doses administered) (18).

Written informed consent was obtained from the parents of each patient and, where possible, from the patient themselves, following a full explanation of the aims of the trial. The study was conducted according to Good Clinical Practice and in compliance with the Declaration of Helsinki with successive amendments. The study protocol was approved by the local Ethics Committee on January 17, 2022 (protocol number: 143 - 21).

### 2.1 Statistical analysis

Numerical variables were expressed as mean and standard deviation (SD); categorical variables were expressed as absolute frequencies and percentage. According to the Kolmogorov-Smirnov test, all variables were normally distributed. The Pearson chi squared test was performed to investigate potential differences in the answers between questionnaire participants (caregiver/patient), gender (male/female), age (pubertal/prepubertal), rate of adherence (good or medium-poor), therapy-related stress (present/absent), and interest in switching to long acting rhGH therapy (present/absent). A univariate regression analysis was carried out to identify which predictors (gender, age, pubertal stage, IQ, parents’ education, employment of the parents, household residence, rhGH therapy duration, stature gain, who administers the therapy, number of weekly injections, perception of therapy’s effectiveness, device characteristics, painful injection, fear of needles, discomfort towards peers related to chronic treatment, problems transporting vials) significantly affect adherence or therapy-related stress. A multivariate regression analysis adjusted for sex, age, pubertal stage, fear of needles, discomfort towards peers related to chronic treatment, was conducted to evaluate predictors of therapy-related stress. For all the statistical analyses, a p value ≤ 0.05 was considered statistically significant. Statistical analyses were per-formed using IBM SPSS for Windows, Version 22 (Armonk, NY, IBM Corp.).

## 3 Results

### 3.1 Features of the population

Seventy patients were recruited: 60% male and 40% female. **Table 1** shows the characteristics of the patients.

**Table 1:** Characteristics of the patients at recruitment.

	Value
N. of patients	70
Gender: *Boys*	42 (60%)
*Girls*	28 (40%)
Puberal Stage: *Prepuberal*	54 (77.1%)
*Puberal*	16 (22.9%)
Diagnosis: *GHD*	48 (68.6%)
*SGA*	22 (31.4%)
Age (years)	11.7 ± 2.86
Therapy duration (months)	67.41 ± 63.22
Height (cm)	139.1 ± 16.7
Height SDS	-1.42 ± 0.81
Weight (kg)	37.4 ± 13.7
Weight (SDS)	-0.69 ± 1.21
BMI (kg/m2)	19.01 ± 4.11
BMI SDS	-0.35 ± 1.35
Growth velocity (cm/years)	6.29 ± 2.57
Growth velocity (SDS)	0.74 ± 2.54
IGF1 (SDS)	1.54 ± 1.46
IQ (points)	94.55 ± 14.79
rhGH dose (mg/kg/day)	0.28 ± 0.34

Data are reported in frequency and percentage or mean value ± SDS. GHD, isolated GH deficiency; SGA, small for gestational age; BMI, body mass index; SDS, standard deviation score, IGF-1, Insulin-like Growth Factor-1; IQ, intellective quotient; rhGH, Recombinant human growth hormone.

The mean age, at the time of questionnaire administration (recruitment) was 11.7 ± 2.86 years; 77.1% were pubertal and 22.9% prepubertal. Among the patients, 68.6% were treated for a diagnosis of isolated GHD, while 31.4% were treated because of SGA. The mean IQ of patients was 94.55 ± 14.79 points at the time of the questionnaire. The median duration of therapy was 49 months (ranging from 12 to 156 months; the mean was 56.2 ± 34.6 months). With regard to the residence of the household, 62.9% of patients lived in a different province from the reference endocrinology center, while 37.1% lived in the same province as the endocrinology center; 74.3% of patients lived in cities and 25.7% in small towns. The household was complete in 85.7%, while in 14.3% it was incomplete. The parents’ level of education was: 36.4% had completed primary school, 52.8% had completed secondary school and 10.0% have a degree. In 55.7% of the households there was at least one homemaker, and in 44.3% of the cases both parents were employed.

### 3.2 Rate of adherence and peculiarities related to drug administration

The questionnaire was completed in 60% of the cases by the patient while in 40% by the parent. **Table 2** shows the response rates to the questionnaire questions. Therapy was administered by the parent or by the patient in 77.1% of cases and 22.9% of cases, respectively. Adherence was good or medium/poor in 82.9% and 17.1% of cases, respectively. rhGH therapy was perceived as useful in 77.1% of cases, while as unhelpful or imposed by parents or doctor in 22.9% of cases.

**Table 2:** Questionnaire questions and answer rates.

Question	Answer options	Frequency [n]	Percentage [%]
Who administers the injection of growth hormone?	Myself	16	22.9%
	Caregivers	54	77.1%
Before beginning growth hormone injections, have you received any training?	Yes	44	62.9%
	No	26	37.1%
How frequently do you have injections of growth hormone?	Daily	48	68.6%
	other	22	31.4%
How many times have you missed an injection in the last six months?	None or < 1 injection missed/week	58	82.9%
	1 to 3 injection missed/week	12	17.1%
	other	0	0%
Why do you think it is useful to regularly perform growth hormone treatment?	because the doctor said it/to make my parents happy	16	22.9%
	to better grow up	54	77.1%
Do you use a ready-to-use pen device?	Yes	40	57.1%
	No	30	42.9%
What do you think of the growth hormone injection?	Easy to do	61	87.1%
	Difficult/painful to do	9	12.9%
Are you scared of needles?	Yes	18	25.7%
	No	52	74.3%
Are you embarrassed with your peers because you have to take a growth hormone injection?	Yes	54	77.1%
	No	16	22.9%
When you go on holiday do you usually take your injection with you?	Yes	67	95.7%
	No	3	4.3%
Are you stressed by the growth hormone injection?	Yes	29	41.4%
	No	41	58.6%
Would you like to participate in a new training course on growth hormone injection?	Yes	31	44.3%
	No	39	55.7%
Would you like to receive further information on the benefits of growth hormone?	Yes	50	71.4%
	No	20	28.6%
What do you think about the possibility of growth hormone therapy once a week rather than every day?	I am interested	51	72.9%
	I am not interested	8	11.4%

Data are reported in frequency and percentage.

With regard to the administration device: the 57.1% of patients had a ready-to-use device, while 42.9% of patients had a device that had to be reconstituted before therapy was administered. The majority of our sample reported no difficulties in preparing and/or administering the injection (87.1% of the participants), while 12.9% reported difficulties. No difficulties with transporting the vials were reported in almost all questionnaires (95.7% of the participants).

All patients and/or caregivers attended a training course at the time the therapy was prescribed. Whereas 44.3% of patients and/or their parents responded positively about the possibility of attending a new training course, 55.7% did not consider a new training course necessary. However, 71.4% of the respondents would like to receive more information about the therapy, compared to 28.6% who did not consider this necessary. Finally, 72.9% were interested in receiving information about long-acting rhGH therapy. Fear of needles was reported in 25.7% of the cases and discomfort towards peers related to chronic treatment in 22.9% of cases. Stress/discomfort related to the therapy was declared in 41.4% of cases; among the subjects reporting stress, 75.8% were pubertal. There were no significant differences in responses with respect to the diagnosis of isolated GHD or SGA (data not showed).

### 3.3 Factors influencing adherence and therapy-related stress

At the univariate regression analysis, adherence was not significantly influenced by gender, age, pubertal stage, IQ, parents’ education, household composition and residence, diagnosis (GHD or SGA), rhGH therapy duration, stature gain, who administers the therapy, number of weekly injections, perception of therapy’s effectiveness, device characteristics, problems transporting vials, painful injection, fear of needles, discomfort towards peers (data not showed).

At the univariate regression analysis, chronic therapy-related stress was significantly influenced by fear of needles (OR 2.9, 95% CI 1.05 - 8.97; p=0.044) and by discomfort towards peers related to chronic treatment (OR 4.4, 95%CI 1.32-14.59; p=0.015) (**Table 3**), while no significant association has been documented with gender, age, pubertal stage, IQ, parents’ education, household characteristics, household residence, diagnosis, rhGH therapy duration, stature gain, who administers the therapy, number of weekly injections, perception of therapy’s effectiveness, device characteristics, problems transporting vials, painful injection (data not showed).

**Table 3:** Univariate and multivariate regression analysis for chronic therapy-related stress.

Variable		Univariate regression	Multivariate regression*
Discomfort towards peers related to chronic treatment	OR	4,40	4,84
	95% IC	1,327 – 14,594	1,30 – 17,99
	*P value*	**0,015**	**0,019**
Fear of needles	OR	2,96	3,18
	95% IC	1.05-8.97	0,91 – 11,08
	*P value*	**0,044**	0,069

*The multivariate regression model included the following variables: discomfort towards peers related to chronic treatment, fear of needles, age, gender, pubertal stage.

Bold values indicate statistically significant p value.

Multivariate regression analysis confirmed the significant influence of discomfort towards peers on chronic therapy-related stress independently of gender, age and pubertal stage (OR 4.84, 95%CI 1.30-17.99; p=0.019), whereas the influence of fear of needles was borderline significant (OR 3.18, 95% CI 0.913 - 11.07; p=0.069) (**Table 3**).

No statistically significant difference was documented between groups with regard to the occurrence of therapy-related stress in relation to who had completed the questionnaire (parent/patient), diagnosis, gender, stage of puberty (pubertal/pre-pubertal) and degree of adherence to therapy. However, it was found that therapy-related stress was reported in 62.1% of the cases when the questionnaire was filled out by the patient, whereas when the filler was the parent, the percentage was 37.9%.

No statistically significant difference was documented between groups with regard to interest in long-acting rhGH regardless of who completed the questionnaire (parent/patient), gender, pubertal stage and degree of adherence, therapy-related stress. However, interest in long-acting therapy stood at 86.3%.

## 4 Discussion

In the present study, a high degree of adherence to rhGH therapy was demonstrated in children and adolescents with GHD or on rhGH therapy because SGA. In fact, 82.9% of patients and/or caregivers who completed the questionnaire reported that they regularly receive and/or administer the treatment prescribed by their physician. This result is in agreement with a part of the scientific data on adherence in pediatric patients on chronic therapy (6), and in particular those on daily rhGH therapy (7).

In a systematic review, Gomez et al. aimed at examining available data on adherence to injectable treatments for chronic diseases in children, with a focus on daily rhGH therapy, found that adherence to rhGH treatment was high (>80%) in many studies, although comparability between studies was limited due to heterogeneity in the definition, measurement and reporting of adherence (6). The high degree of adherence to therapy in pediatric age could be favored by parental support in the management of treatment. However, the degree of adherence may vary depending on the methodology used for the assessment, and in some cases may be low even in pediatric age (7, 19, 20). Consistently, the systematic review by Fisher et al. found that non-adherence ranged from 5% to 82%, depending on the methods and definitions of adherence applied (7). In a multicenter study, Bagnasco et al. reported that 24.4% of children and adolescents on daily rhGH therapy were non-adherent (as they missed one or more injections during the week), and in particular adolescents were 63% less likely to adhere to rhGH therapy than prepubertal children (20). In addition, it is not uncommon for patients to overestimate their level of adherence to treatment (21). To date, there is no univocal definition of non-adherence with respect to the number of weekly and/or monthly non-adherence administrations.

Suboptimal adherence to daily rhGH therapy may result in impaired statural outcome (7, 22, 23). A recent multicenter observational study, the ECOS study, documented that in a population of children treated with rhGH, height recovery over years of therapy were directly correlated with adherence (23). These results were confirmed by other research, which documented better height gain in patients with good adherence than in those with poor adherence (18, 24).

Therefore, the assessment of adherence in children treated with daily rhGH therapy is essential to identify the presence and reasons for poor adherence promptly, and to implement corrective measures so that adherence may improve. Several factors, both modifiable and non-modifiable, may be connected to non-adherence, including low parental education, long duration of the treatment, dissatisfaction with treatment response, insufficient knowledge of the disease and treatment, fear of needles, difficulty in administering the drug, insufficient contact with the physician (25–28). On the other hand, factors that have been linked to higher adherence rates include the utilization of easy-to-use drug delivery devices and pre-filled injection devices, the use of thinner needles, and the awareness of the negative effects of nonadherence (6, 7, 10, 13).

In our population, none of the several factors analyzed had significantly influenced the degree of adherence, which was found very high. However, the method of assessing adherence by means of questionnaires does not entirely exclude confounding factors influencing the completion of questionnaires, including the ‘white coat’ effect, difficulty recalling details of medication administration, desire to avoid confrontation, fear of disappointing clinicians or a combination of reasons (7).

Another aspect to be taken into account is that, even in the presence of a good degree of adherence, the child and especially the adolescent may negatively experience the chronic daily therapy in which they do not feel fully involved. Consistently, when the initiation of therapy occurs in childhood, under close parental supervision, this may result in a lower level of treatment awareness in the patient who will continue therapy into adolescence, during which period the patient may experience treatment negatively if he or she does not feel sufficiently involved. In our study, in 41.4% of the questionnaires, a feeling of stress related to chronic treatment emerged. Stress associated with daily chronic therapies, including rhGH, might have a negative impact on the QoL of children and their caregivers (12, 16), although stress is a condition that is often difficult to objectify and may not emerge in tests commonly used to assess QoL, defined as “*the patient’s perception of the impact of illness and treatment, functioning in a variety of dimensions including physical, mental and social domines”* (7).

In a recent Italian survey study included 142 children/adolescents with GHD and their parents, overall high health-related generic quality of life (HRQoL) was documented in treated GHD patients, comparable to that of healthy people. Similarly, the authors documented good results, comparable to that of international reference values for patients with GHD/Idiopathic short stature, by the Quality of Life in Short Stature Youth (QoLISSY). However, evaluating the data from the different domains, the authors pointed out that GHD children’s scores were lower than the GHD-specific reference values for all domains (social, emotional), with the exception of the physical domain. In relation to the GHD-specific reference values, parents’ scores were found to be lower in the social, emotional, therapeutic, parental effects, and overall score domains (29). Backeljauw et al. in a systematic review, which aimed to assess QoL in parents and children with predominantly idiopathic short stature, SGA, GHD, stated that of the 33 studies examined, many reported significantly worse QoL than children with normal stature (30). However, it was also observed that children receiving rhGH and achieving a good stature gain showed improvements in many aspects of their QoL, including the emotional scale (31).

Narrative Medicine used to evaluate the experiences of patients and caregivers involved in chronic rhGH therapy showed that, despite a high level of awareness of the importance of regular therapy, revealed signs of distress, from feelings of impatience to intolerance, especially on the part of adolescents, regarding chronic daily treatment (32). Adolescents are often in search of greater autonomy and awareness with regard to therapy, while feelings of fear and suffering over injections prevail in children (32, 33). In our study it was documented that more than 50% of the cases where therapy-related stress was reported on the questionnaire were completed by adolescents (62.1%), compared to less than 50% of the questionnaires completed by parents (37.9%). Although this difference did not reach statistical significance, this finding suggests that the adolescent age, when peer interaction increases, may be at higher risk of developing therapy-related stress than the childhood age.

In our research possible factors influencing therapy-related stress in patients and caregivers were analyzed. Fear of needles and discomfort towards peers significantly appear to influence therapy-related stress. In particular, discomfort with peers emerged as the most important factor influencing therapy-related stress, irrespective of gender, age, pubertal stage and fear of needles. Discomfort towards peers may be caused both by organizational implications related to the need for daily therapeutic administrations, but also by the condition of short stature often related to feelings of anxiety, depression, social isolation and difficulties maintaining attention (28, 29). Consistently, Aryayev et al. demonstrated a higher frequency of ‘emotional and peer problems’ and lower self-esteem in children with GHD compared to healthy children, which ultimately resulted in psychosocial maladjustment and conceptualization of internalizing problems in children with GHD (32).

Our study revealed that, even under conditions of high adherence rates, it is possible to detect emotional distress in children and adolescents treated with daily rhGH administrations which, if not explored, may not clearly emerge during medical assessments and possibly affect adherence and thus the effectiveness of treatment. As a vicious circle, the poor response to treatment may further worsen the patient’s feelings of discomfort and low self-esteem. Therefore, psychological support in pediatric patients chronically treated with rhGH and their caregivers plays a crucial role in patient care and support for caregivers.

Moreover, another aspect that should not be underestimated in this context is the emotional burden and stress that caregivers experience in dealing with their child’s chronic illness. Assessing aspects of the parent/caregiver’s care burden is crucial as it is closely related to the well-being of the child/adolescent with a chronic illness and the caregiver themselves. In a review of the literature, Lackener et al. documented that, through different assessment methods (mainly questionnaires), several scientific papers emphasized the presence of caregivers’ burdens, anxiety and stress mainly related to logistical problems in the management of their children’s chronic daily rhGH therapy, social stigmatization and difficulties in comparing with peers, and anxiety about the child’s future prospects in society; parents’ stress was often correlated with the children’s degree of psychosocial functioning (34).

In this study, interest in the possibility of long-acting GH therapy emerged in almost all subjects, independently of who filled out the questionnaire. This result suggests that this therapeutic possibility could be one of the ways to alleviate the emotional burden of chronically treated rhGH pediatric patients and their caregivers.

Our research has some limitations. The study included limited sample sizes. No specific assessment of the patients’ psychosocial functioning was carried out. Among the limitations may be considered the intrinsic limitation of the questionnaire method during the completion of which the patient/caregivers may not be completely objective due to the ‘white coat’ effect or the fear of disappointing clinicians or a combination of reasons. It was not possible to stratify adherence into three grades (high, medium, low), but only into two grades, due to the low number of poorly adherent patients.

On the other hand, our study has significant strengths. Our study population consisted of a homogeneous sample of Caucasian children on rhGH therapy with equal distribution according to sex; all patients had been on rhGH therapy for at least one year and were regularly followed up at the Clinical Centre. In addition, the inclusion of patients diagnosed with GHD or SGA who were not on other therapies and who were not affected by other diseases limited the possible influence of other factors on the results. All recruited subjects were investigated for IQ, which was within normal limits throughout the sample. Finally, the completion of the questionnaires was conducted under the supervision of a psychologist who provided the necessary support including relieving patients and caregivers of any burdens associated with completing the questionnaire.

## 5 Conclusions

Daily rhGH therapy, even in the presence of a high degree of adherence, may burden the patient’s psychological well-being for the pediatric patient and caregivers, although this discomfort is not always clearly evident. Discomfort towards peers related to chronic treatment was found to be associated to stress related to daily rhGH therapy in children and adolescents. All factors that may influence the well-being of the patient in chronic therapy should be prospectively identified in order to set up appropriate strategies to ensure that they do not adversely affect the quality of life of rhGH treated children and adolescents.

## Data Availability

The raw data supporting the conclusions of this article will be made available by the authors, without undue reservation.
